# Upregulation of miR-126-3p promotes human saphenous vein endothelial cell proliferation *in vitro* and prevents vein graft neointimal formation *ex vivo* and *in vivo*

**DOI:** 10.18632/oncotarget.22365

**Published:** 2017-11-03

**Authors:** Qingxi Qu, Weidong Bing, Xiangbin Meng, Jie Xi, Xiao Bai, Qing Liu, Yaqiu Guo, Xin Zhao, Yanwen Bi

**Affiliations:** ^1^ Department of Cardiovascular Surgery, Qilu Hospital of Shandong University, Jinan, Shandong 250012, China; ^2^ Department of Ultrasound, Shandong Provincial Hospital Affiliated to Shandong University, Jinan, Shandong 250012, China; ^3^ Department of Anesthesiology, Jinan Maternity and Child Health Care Hospital, Jinan, Shandong 250012, China

**Keywords:** miRNAs, human saphenous vein, vein graft, endothelial cells, neointimal formation

## Abstract

Poor long-term patency of vein grafts remains an obstacle in coronary artery bypass grafting (CABG) surgery using an autologous saphenous vein graft. Recent studies have revealed that miR-126-3p promotes vascular integrity and angiogenesis. We aimed to identify the role of miR-126-3p in the setting of vein graft disease and investigate the value of miR-126-3p agomir as a future gene therapy in vein graft failure. Expression analysis of circulating miR-126-3p in plasma from CABG patients established the basic clues that miR-126-3p participates in CABG. The *in vitro* results indicated that elevated miR-126-3p expression significantly improved proliferation and migration in human saphenous vein endothelial cells (HSVECs) by targeting sprouty-related protein-1 (SPRED-1) and phosphatidylinositol-3-kinase regulatory subunit 2 (PIK3R2), but not in human saphenous vein smooth muscle cells (HSVSMCs). Moreover, the therapeutic potential of miR-126-3p agomir was demonstrated in cultured human saphenous vein (HSV) *ex vivo*. Finally, local delivery of miR-126-3p agomir was confirmed to enhance reendothelialization and attenuate neointimal formation in a rat vein arterialization model. In conclusion, we provide evidence that upregulation of miR-126-3p by agomir possesses potential clinical value in the prevention and treatment of autologous vein graft restenosis in CABG.

## INTRODUCTION

Coronary artery disease (CAD) is one of the leading causes of morbidity and mortality according to a report from the World Health Organization. Despite the increased utility of drug-eluting stents (DES), CABG remains a unique treatment of choice in patients with multi-vessel disease, occlusive arterial disease or patients with multiple risk factors for repeat intervention [1–3]. Autologous saphenous veins is the most common type of conduit grafts for bypass grafting, and revascularization with saphenous vein grafts is a standard surgical therapy for CABG [4]. However, the benefits of CABG surgery remain limited by the life expectancy of the saphenous vein graft, as over 50% of saphenous vein grafts will be occluded during the first decade after surgery, leading to recurrent symptoms, myocardial infarction or reoperative revascularization [5]. The principal process responsible for vein graft failure occurs as a result of massive neointimal hyperplasia, which takes place in response to vessel wall injury (distention, trauma) and hemodynamic changes, and is characterized by deposition of vascular smooth muscle cells (VSMCs) and extracellular matrix (ECM) components, such as proteoglycans and collagens [6]. Our previous study demonstrated that selective activation of reendothelialization after vein grafting attenuated neointimal lesion formation [7]. The proliferative and migratory activity of endothelial cells (ECs) are commonly recognized to play a central role in the promotion of reendothelialization and restoration of endothelial function [8, 9]. Therefore, specific approaches to selectively accelerate dysfunctional endothelial recovery of saphenous vein grafts are promising methods to further improve the therapeutic effect of CABG.

MicroRNAs (miRNAs, miRs) are endogenous small non-coding RNAs that regulate diverse cellular processes, such as proliferation, differentiation, and migration, by binding to target mRNAs and inducing their translational repression or degradation [10, 11]. Multiple lines of *in vitro* and vivo experimental studies have shown that miRNAs are dysregulated in response to acute or chronic vascular injury and that modulation of miRNA levels may serve as an attractive therapeutic target to improve outcomes of patients with vascular diseases [12–17]. Recently, miR-126-3p has emerged as irreplaceable endothelial cell–restricted miRNA that is involved in the regulation of vascular integrity and angiogenic signaling [18, 19]. The roles of miR-126-3p are often discussed in the context of a number of vascular diseases including myocardial infarction, diabetic vascular complications, pulmonary arterial hypertension, and ischemic stroke [20–23]. There is a growing body of evidence suggesting that miR-126-3p may play a vasculoprotective role in vein graft disease [24–28].

To the best of our knowledge, no studies to date have investigated the role of miR-126-3p associated with vein graft disease. Whether miR-126-3p could be used as an intervention to prevent vein graft failure remains uncertain. In the present work, we compared the expression levels of miR-126-3p in patients with CAD before and after CABG. To investigate the role of miR-126-3p in vein graft cells, we isolated HSVECs and HSVSMCs from fresh HSV segments that were obtained from CABG patients. We also established the ex vivo HSV culture model and rat vein grafting model to better assess whether a single delivery of miR-126-3p agomir would be sufficient to reduce intimal hyperplasia of the vein graft. The ultimate goal of our study was to investigate the role of miR-126-3p in vein graft disease and the value of the miR-126-3p agomir as a future gene therapy in CABG.

## RESULTS

### Altered miR-126-3p expression in patients with CAD after CABG

**Figure 1:**
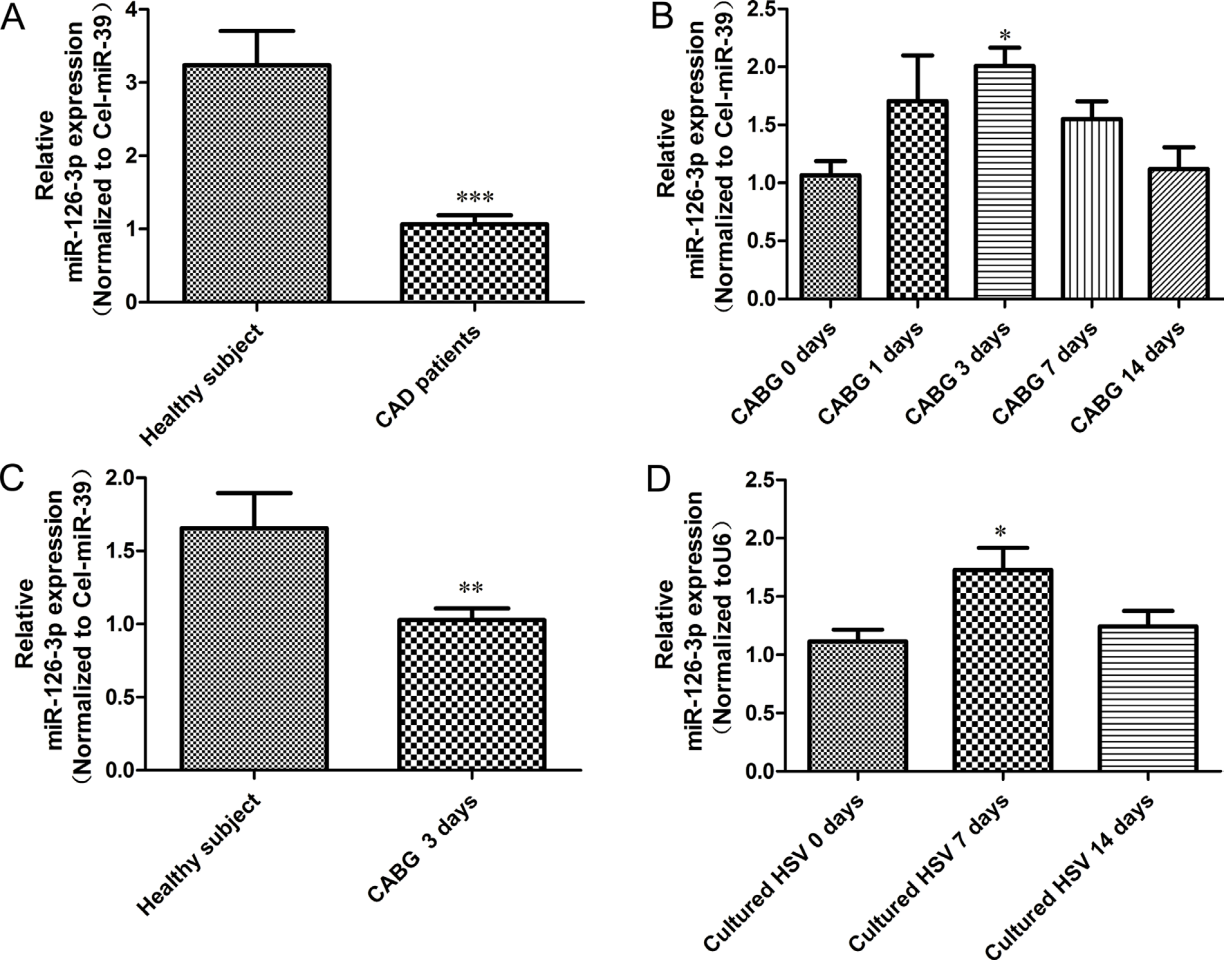
Relative expression levels miR-126-3p in plasma from CABG patients and vein graft model (**A**) Circulating miR-126-3p expression was detected by real-time PCR in human plasma samples from healthy controls and patients with complex multi-vessel coronary artery disease prepping for a CABG. (**B**) Constant, dynamic changes of circulating miR-126-3p expression in patients at different time points of CABG demonstrating upregulation of miR-126-3p in plasma after surgery. (**C**) Differential expression of miR-126-3p in the plasma of normal individuals and CABG patients following grafting for 3 days. (**D**) Real-time PCR to assess miR-126-3p expression in human saphenous veins subjected to culture for a period of 0, 7, and 14 days to allow neointimal lesion development. Values are expressed as the mean ± SEM. *n =* 10. Significance levels, *P < 0.05; **P < 0.01; ***P < 0.001.

To investigate the role of miR-126-3p in the clinical setting of vein graft disease, we first evaluated the expression of plasma miR-126-3p in CAD patients by preparing CABG and healthy volunteers from the medical examination center of our hospital by qRT-PCR. Compared with healthy subjects, all preoperative patients had a significantly lower level of miR-126-3p expression (Figure 1A, *P* < 0.0001). To further validate the relevance of miR-126-3p in CABG, we assessed dynamic changes in plasma miR-126-3p expression in patients undergoing CABG at different time points during the perioperative period. Circulating miR-126-3p levels were continuously detected by qRT-PCR 1 day prior to surgery and at 1, 3, 7 and 14 days after CABG. We found that circulating miR-126-3p levels in patients undergoing CABG showed a brief rise during the perioperative period, which increased one day after surgery, peaked at 3 day after surgery, remained up-regulated at 7 days after surgery, and then gradually declined to baseline at 14 days after surgery (Figure 1B, *P* < 0.05). However, even the peak value remained far below the levels of miR-126-3p expression in normal people (Figure 1C, *P* < 0.01). Thus, we hypothesize that the transient change in circulating miR-126-3p in CABG surgery may reflect early compensatory mechanism by which endothelial cell function is maintained in vein grafts, but this compensation is incomplete.

To further test this assumption, we established a HSV organ culture model to validate the role of miR-126-3p in neointimal formation of vein grafts. Discarded HSVs were obtained from patients receiving CABG surgery and cultured for 7 and 14 days ex vivo. RT-PCR revealed that the miR-126-3p level was increased by 55.2% (*P* < 0.05) in vein segments cultured for 7 days compared with segments that were not cultured (Figure 1D). Although miR-126-3p was still up-regulated at 14 days, there were no significant differences compared with those that were not cultured. In general, the changes in miR-126-3p in HSV were almost consistent with the findings obtained for human plasma.

### Effect of miR-126- 3p overexpressionon adult HSVEC proliferation and migration *in vitro*

**Figure 2:**
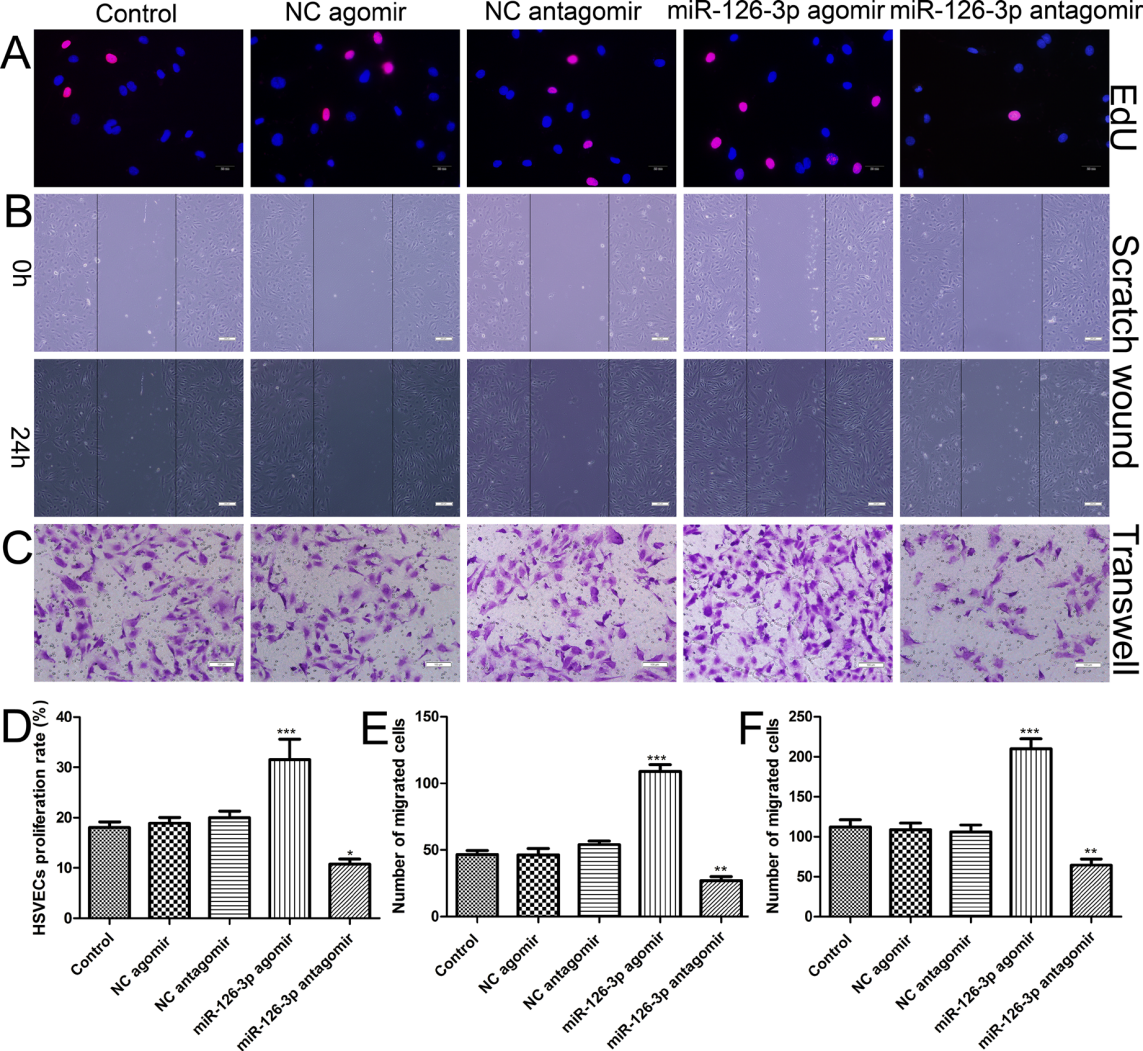
Effect of miR-126-3p on the proliferation and migration of HSVECs HSVECs were transfected with 100 nmol miR-126-3p agomir and antagomir. (**A**) Representative images of the EdU incorporation assay of HSVECs. EdU-positive cells were red-stained, and negative cells had blue nuclei (Hoechst 33342). Scale bars represent 20 μm. (**B**) Representative images of cell migration after scratching in miR-126-3p agomir-transfected HSVECs. Scale bars represent 200 μm. (**C**) Representative images of the transwell assay of in miR-126-3p agomir-treated HSVECs. Scale bars represent 100 μm. (**D**) Quantification of proliferation rates (percentage of EdU-positive cells) of HSVECs. (**E**) The numbers of cells that migrated to the wound zones of the scratch wound assay were quantified. (**F**) The mean number of migrated cells was quantified. The results are presented as the mean ± SEM from 3 independent experiments. *P < 0.05; **P < 0.01; ***P < 0.001 vs control.

To investigate the function of miR-126-3p in vein graft cells and provide evidence for further applications, we successfully isolated and identified primary HSVECs from patients undergoing CABG surgery (Supplementary Figure 1). At 24 h after Cy3-labeled miR-126-3p agomir transfection in HSVECs, specific red fluorescence could be detected within the cytoplasm at concentrations ranging from 25 to 200 nmol (Supplementary Figure 2). The results suggested that, in theory, exogenous agomirs entered the cells and could exert their biological functions. Because the 100 and 200 nmol agomir groups clearly showed an equivalent high fluorescence intensity during transfection, we selected the 100 nmol agomir for the following EC transfection studies. HSVECs were transfected with miR-126-3p agomir, miR-126-3p negative control agomir (NC agomir), miR-126-3p antagomir and miR-126-3p negative control antagomir (NC antagomir). As shown in Figure 2A using the EdU incorporation assay, the purple nuclei are EdU positive and indicate proliferating cells. Overexpression of miR-126-3p resulted in an increased proliferation rate, whereas inhibition of miR-126-3p had the opposite effect (Figure 2D). We continued to assess whether miR-126-3p had the ability to increase HSVEC migration. The results of the scratch wound assay (Figure 2B) and the transwell migration assay (Figure 2C) showed that the migration capacity of cells transfected with the miR-126-3p agomir was dramatically increased compared with the NC agomir and control group, and the migration capacity of cells transfected with the miR-126-3p antagomir was dramatically decreased compared with the NC antagomir and control group cells (Figure 2E and 2F). Taken together, these data suggested that miR-126-3p participates in HSVEC proliferation and migration and that agomir treatment is a viable gene strategy to increase miR-126-3p functions in HSVECs.

### The levels of two target genes were altered after miR-126-3p treatment

**Figure 3:**
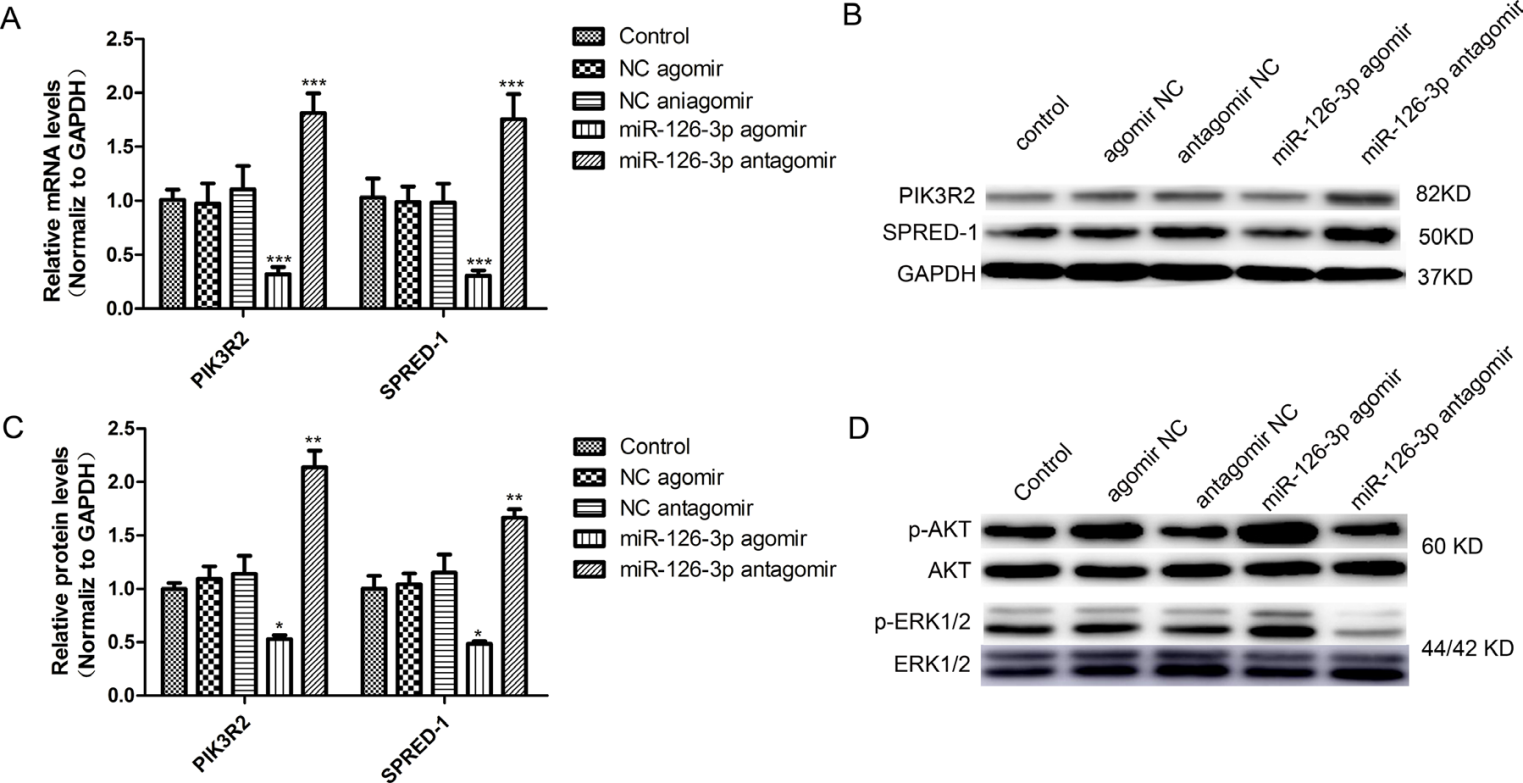
miR-126-3p targeted PIK3R2 and SPRED-1 and activated the ERK1/2 and AKT signaling pathways in HSVECs (**A**) Overexpression and inhibition of miR-126-3p, respectively, promoted and inhibited PIK3R2 and SPRED-1 mRNA levels in HSVECs. (**B**) Representative western blots of PIK3R2 and SPRED-1 protein expression after transfection of miR-126-3p agomir and antagomir in HSVECs. (**C**) Western blotting analyses confirmed the downregulation and upregulation of PIK3R2 and SPRED-1 proteins in HSVECs by the miR-126-3p agomir or antagomir, respectively. (**D**) Representative western blots of the upregulation and downregulation of AKT phosphorylation and ERK1/2 phosphorylation in HSVECs by the miR-126-3p agomir or antagomir, respectively. The results are presented as the mean ± SEM from 3 independent experiments. *P < 0.05; **P < 0.01; ***P < 0.001 vs control.

Among the verified targets of miR-126-3p, SPRED-1and PIK3R2 are known as negative regulators of the VEGF (vascular endothelial growth factor) signaling pathway to critically influence proliferation and migration in human umbilical vein endothelial cells (HUVECs) [24]. We assessed whether the genes and pathways were involved in HSVECs. To test this hypothesis and confirm the previous results, cultured HSVECs were transfected with either miR-126-3p agomir or miR-126-3p antagomir. As shown in Figure 3A, miR-126-3p overexpression and inhibition were, respectively, responsible for downregulation (0.47- and 0.51-fold; *P* < 0.001) and upregulation (1.14- and 1.67-fold; *P* < 0.001) of the PIK3R2 and SPRED-1 mRNA levels. Consistent with these findings, the expression of both PIK3R2 and SPRED-1 proteins in HSVECs was significantly decreased upon treatment with the miR-126-3p agomir and was significantly increased upon transfection with the miR-126-3p antagomir (Figure 3B and 3C). It is well established that activation of the ERK1/2 and AKT signaling pathways is involved in VEGF stimulation of endothelial cell proliferation and migration [29]. Notably, HSVEC treatment with miR-126-3p agomir resulted in increased ERK1/2 and AKT phosphorylation, whereas transfection with the miR-126-3p antagomir had the opposite effect (Figure 3D). Taken together, these results implied that overexpression of miR-126-3p by agomir in HSVECs promoted proliferation and migration mediated, at least in part, by the regulation of ERK1/2 and AKT signaling through SPRED-1 and PIK3R2 targeting.

### miR-126-3p had no effect on HSVSMC proliferation and migration *in vitro*

**Figure 4:**
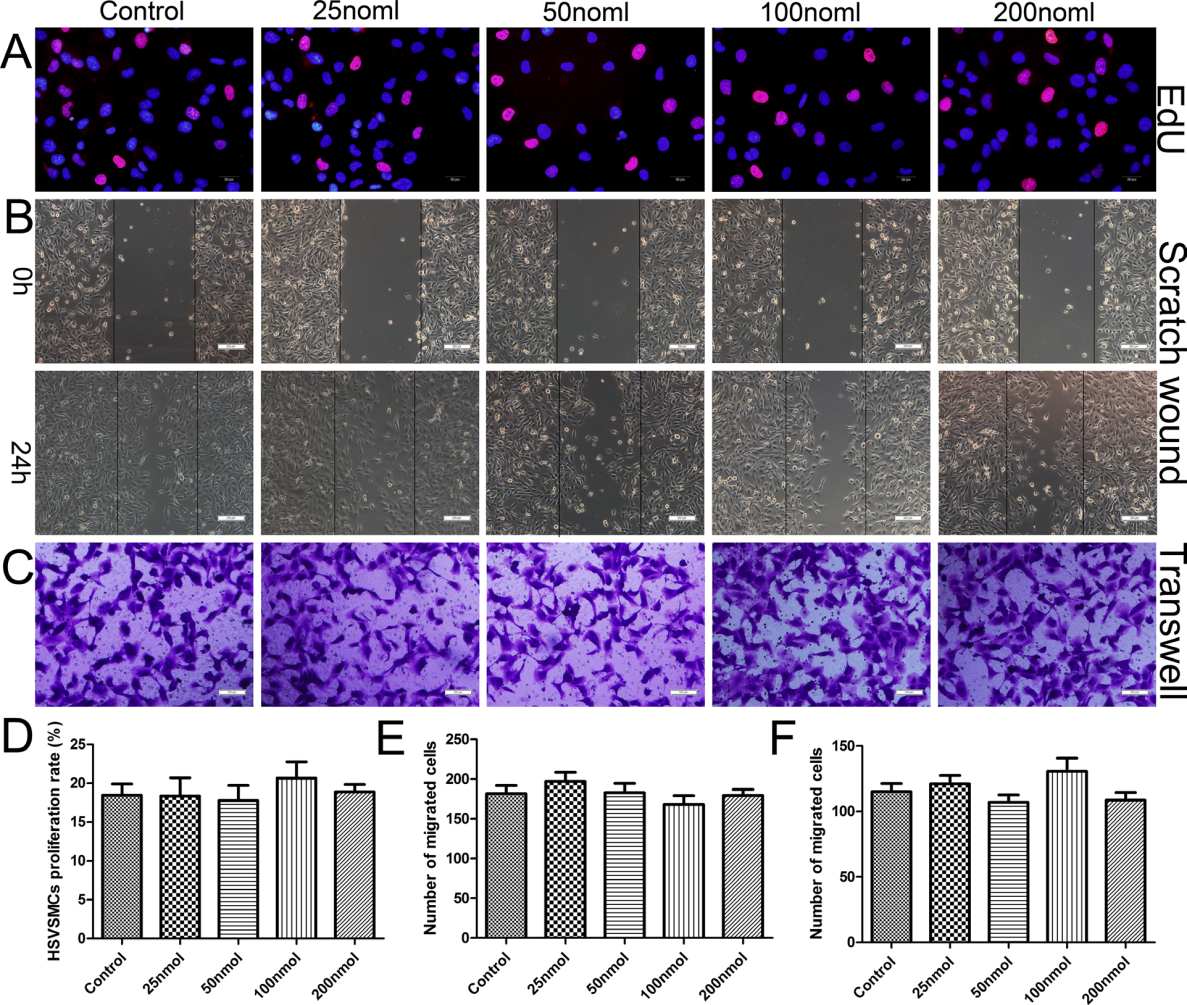
miR-126-3p agomir had no effect on HSVSMC proliferation and migration *in vitro* (**A**) Representative images of the EdU incorporation assay of HSVSMCs treated with 25, 50, 100 or 200 nmol miR-126-3p agomir. EdU-positive cells were red-stained, and negative cells had blue nuclei (Hoechst 33342). Scale bars represent 20 μm. (**B**) Representative images of cell migration after scratching in miR-126-3p agomir-transfected HSVSMCs. Scale bars represent 200 μm. (**C**) Representative images of the transwell assay of miR-126-3p agomir-treated HSVSMCs. Scale bars represent 100 μm. (**D**) Quantification of the proliferation rates (percentage of EdU-positive cells) of HSVSMCs. (**E**) The numbers of cells that migrated to the wound zones of the scratch wound assay were quantified. (**F**) The mean of number of migrated cells was quantified.

To examine the side effects of dysregulated miR-126-3p expression in SMCs of vein grafts, primary HSVSMCs were isolated and identified (Supplementary Figure 3). Loss-of-function studies were not performed in this part of the analysis because no previous evidence have suggested that miR-126-3p is endogenously expressed in VSMCs. First, we examined the effect of transduction with the miR-126-3p agomir on the proliferation of HSVSMC by the EdU incorporation assay; however, 25–200 nmol of miR-126-3p agomir did not promote SMC proliferation (Figure 4A and 4D) compared with the control. Next, we investigated the behavior of SMC migration following transduction using a scratch wound assay (Figure 4B) and transwell migration assay (Figure 4C). Studies have shown that the upregulation of EC-specific miR-126-3p by agomir has no influence on basal HSVSMC migration (Figure 4E–4F). Finally, we generated an inflammatory cytokine-induced inflammation model *in vitro* [30], in which HSVSMCs were cultured with tumor necrosis factor-α (TNF-α, 10 ng/ml) and increasing concentrations of miR-126-3p agomir for the tested functions. Similarly, miR-126-3p agomir at all tested concentrations ranging from 25–200 nmol did not activate HSVSMC proliferation or migration induced by the inflammatory mediator compared with the control (Supplemental Figure 4). Taken together, these data demonstrated that the effect of miR-126-3p on cell migration and proliferation is cell type-specific and that the miR-126-3p agomir had no effect on HSVSMC proliferation and migration.

### Overexpression of miR-126-3p by agomir inhibited neointimal formation in HSV organ cultures *ex vivo*

**Figure 5:**
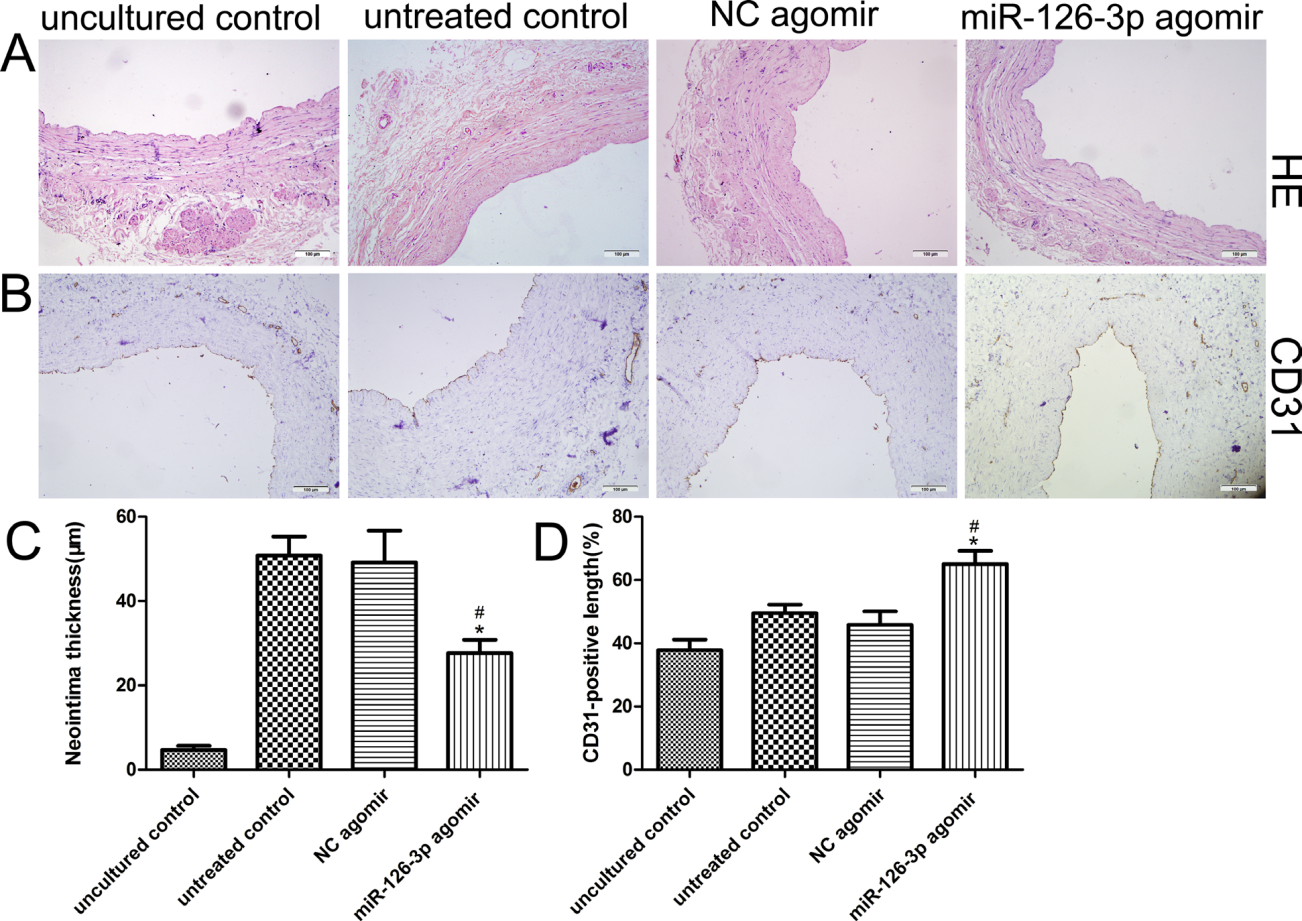
Effect of the miR-126-3p agomir on the human saphenous vein organ culture model (**A**) Representative HE stained cross-sections of HSV segments after 14 days of culturing *ex vivo*. (**B**) Representative CD31 immunohistochemical staining cross-sections of HSV segments after 14 days of culturing *ex vivo*. (**C**) Quantification of the neointimal thickness of independently cultured HSVs. (**D**) Quantification of the ratio of the CD31 length to lumen perimeter in sections of independently cultured saphenous veins. Scale bars represent 100 μm. Values are expressed as the mean ± SEM. *n =* 6. ^#^*P* < 0.001 vs uncultured vein, *P < 0.01 vs NC agomir-treated and miR-126-3p agomir-treated veins.

Based on our clinical findings and *in vitro* results indicating the beneficial effects of miR-126-3p on HSVEC proliferation and migration, we hypothesized that overexpression of miR-126-3p by agomir could inhibit neointimal hyperplasia by accelerating reendothelialization in a well validated *ex vivo* organ culture model of HSV. After culturing *ex vivo* for 14 days, HSV segments were either not transfected or were transfected with miR-126-3p agomir, resulting in evident neointimal hyperplasia compared with the uncultured samples (Figure 5A). The morphometric analysis showed that neointimal thickening was significantly attenuated in miR-126-3p agomir-transfected veins compared with NC agomir-transfected veins and untreated veins (27.67 ± 3.16 μm vs 49.17 ± 7.56 μm and 50.83 ± 4.5 μm, respectively, *P* both < 0.01 Figure 5C). Immunohistochemical staining revealed coverage of the luminal surface with CD31-positive cells 2 weeks after culture (Figure 5B), and immunohistochemical analysis demonstrated that treatment with the miR-126-3p agomir increased the percentage of endothelial cell coverage at day 14 compared with the cultured controls, but delivery of the miR-126-3p NC agomir did not exhibit this effect (65 ± 4.27% vs 45.83 ± 4.28% and 49.5 ± 2.73%, respectively, *P* both < 0.01, Figure 5D).

### Local delivery of miR-126-3p agomir improved blood flow *in vivo i*n a rat vein grafting model

**Figure 6:**
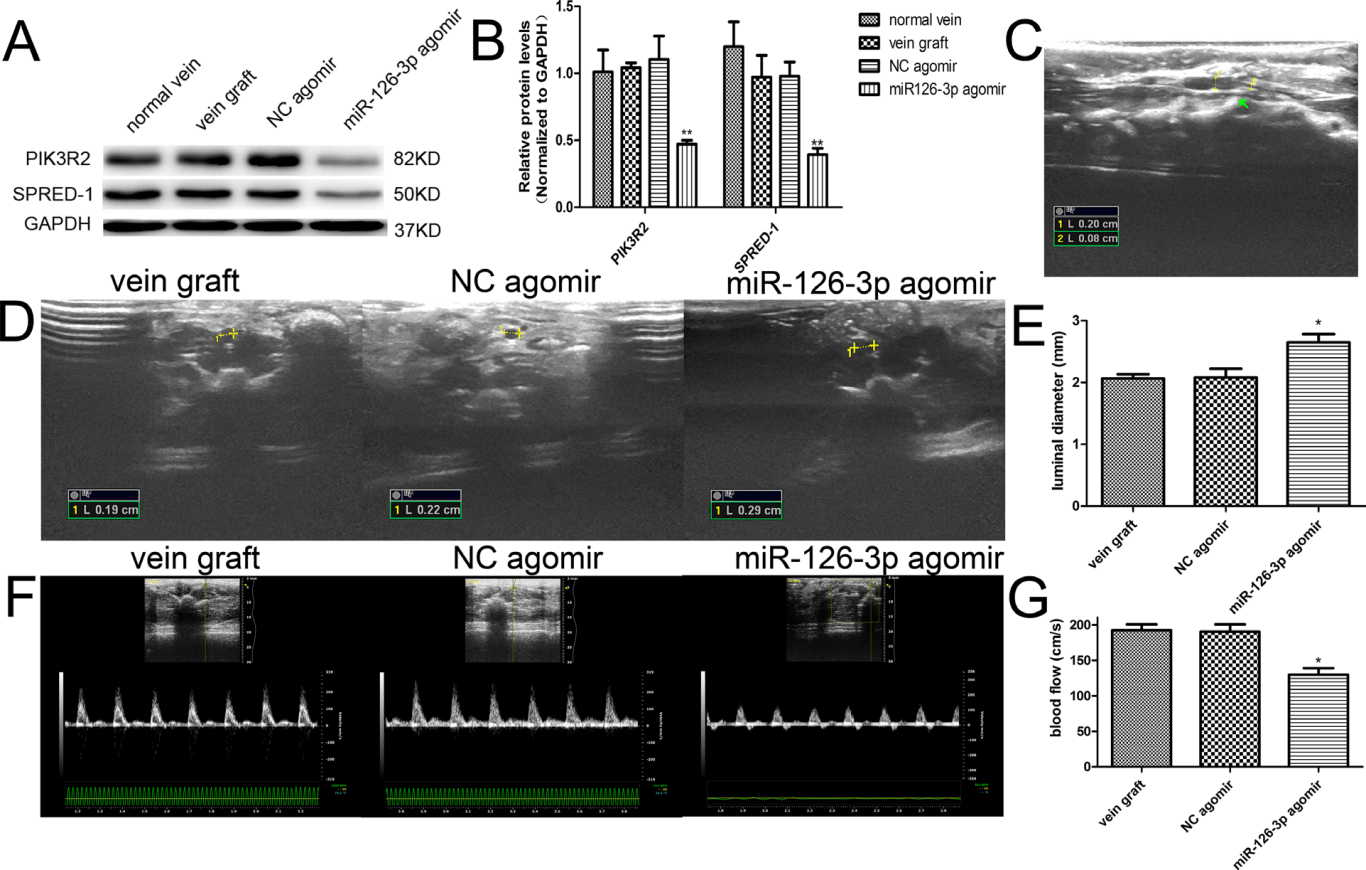
Exogenous miR-126-3p improved blood flow in vein grafts *in vivo* (**A**) Representative western blots and quantification (**B**) of PIK3R2 and SPRED-1 proteins are shown. PIK3R2 and SPRED-1 protein levels were significantly downregulated by local delivery of the miR-126-3p agomir at 7 days after transfection. (**C**) The vein grafts were all patent during the whole observation period, and longitudinal imaging emphasizes the internal diameter of the cannula and the vein graft at 4 weeks after surgery. (**D**) Representative ultrasound images and quantification (**E**) of the luminal diameter in vein grafts at 28 days after surgery are shown. (**F**) Representative ultrasound images and quantification (**G**) of the peak systolic velocity in vein grafts at 28 days after surgery are shown. Values are expressed as the mean ± SEM. *n =* 3–6 per group. *P < 0.05, **P < 0.01 vs NC agomir and vein graft groups.

Owing to the limitations of the *ex vivo* model, we performed further experiments to test the feasibility of applying miR-126-3p agomir *in vivo* and provide a practical basis for further clinical application. We established a rat vein arterialization model and adopted local overexpression of miR-126-3p in vein bypass grafts prior to implantation using a “cuff” anastomotic technique (Supplementary Figure 5). As shown in Supplementary Figure 6, transfected vein grafts showed broadly apparent Cy3 staining throughout the vessel wall under fluorescent light at 24 hours after Cy3-labeled miR-126-3p agomir transfection, and it was still detectable after 7 days. None of the remote organs, such as the heart, lungs, liver and kidneys, displayed evidence for Cy3-labeled agomir expression (data not shown). Western blot analysis indicated that local delivery of the miR-126-3p agomir resulted in significantly downregulated protein levels of PIK3R2 and SPRED-1 at 7 days after surgery (Figure 6A and 6B), which also supported that the local delivery of miR-126-3p agomir prior to implantation was efficient and that PIK3R2 and SPRED-1 were indeed target genes of miR-126-3p *in vivo*.

The use of vascular ultrasound enabled us to noninvasively evaluate graft vascular patency. The implanted grafts were all patent during the whole observation period (Figure 6C). Meanwhile, the vascular diameter and blood flow in the distal anastomosis of vein grafts were measured and analyzed 4 weeks after surgery. The luminal diameter of vein grafts either in the NC agomir group or vein graft group was thinner than that in the miR-126-3p agomir treated group (2.08 ± 0.14 mm and 2.07 ± 0.07 mm vs. 2.65 ± 0.13 mm, respectively, *P* both < 0.05, Figure 6D and 6E). Consistent with the findings for the vascular diameter, the peak-systolic velocity was significantly lower in the miR-126-3p agomir-treated group than in the NC agomir group and vein graft group (130 ± 9.31 cm/s vs. 190.8 ± 10.2 cm/s and 192.5 ± 8.4 cm/s, respectively, *P* both < 0.05, Figure 6F and 6G). There were no significant difference between the NC agomir group and the vein graft group.

### Therapeutic upregulation of miR-126-3p by agomir attenuated vein graft neointimal formation *in vivo*

**Figure 7:**
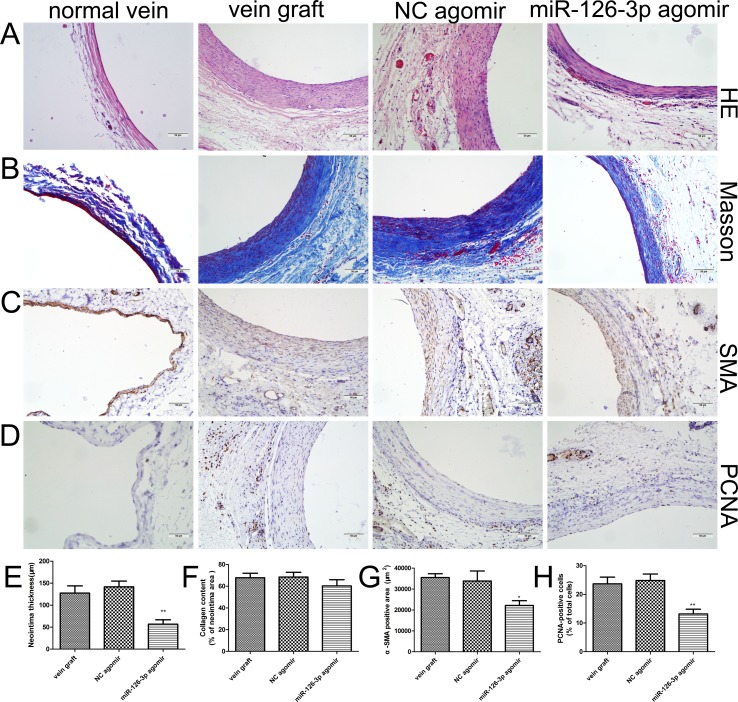
miR-126-3p overexpression limited neointimal formation of vein grafts *in viv*o (**A**–**D**) Representative photomicrographs of vein grafts from different groups at 28 days after surgery. Sections were processed for HE staining to measure the neointimal thickness (A), Masson trichrome staining to analyze collagen (B), immunohistochemistry for α-SMA to detect vascular smooth muscle cells (C), or PCNA immunostaining to identify vascular proliferating cells (D). (**E**–**H**) Bar graphs depicting the quantitative analysis of the neointimal thickness (E), the percentages of collagen (F), α-SMA (G) and PCNA (H)-positive cells in the neointima. Values are expressed as the mean ± SEM. *n =* 6 per group. *P < 0.05, **P < 0.01 vs NC agomir and vein graft groups. Scale bars represent 50 μm.

Histomorphometric analysis was performed at 4 weeks after venous implantation (Figure 7A–7D). Hematoxylin eosin staining revealed that treatment with miR-126-3p agomir dramatically reduced neointimal thickness, whereas treatment with NC agomir and no treatment resulted in a dramatically increased neointima (56.83 ± 9.99 μm vs 141.7 ± 13.12 μm and 127.3 ± 16.64 μm, respectively, *P* both < 0.01, Figure 7A and 7E). These results were consistent with the findings obtained for cultured HSVs *ex vivo*. To gain further insights into the observations mentioned above, we performed histochemistry for collagen by Masson trichrome staining (Figure 7B) and immunohistochemistry for SMCs using α-SMA antibodies (Figure 7C). The Masson trichrome staining showed that the percentage of neointimal area occupied by blue-colored fibers (collagen) did not obviously differ between the miR-126-3p agomir-treated group and the other control groups *(P* > 0.05, Figure 7F). Moreover, immunohistochemical staining of α-SMA demonstrated the presence of abundant SMCs localized in the neointima in vein grafts, and the accumulation of positively stained SMCs was markedly reduced in vein grafts treated with miR-126-3p agomir (22160 ± 2271 mm^2^ vs 33832 ± 4735 mm^2^ and 35468 ± 1803 mm^2^, respectively, *P* both < 0.05, Figure 7G). Moreover, we also conducted staining with proliferating cell nuclear antigen (PCNA) to identify vascular proliferating SMCs after venous implantation. Consequently, almost no expression of PCNA protein was detected in the normal vein, but PCNA-positive cells were prominent in the neointima of arterialized veins (Figure 7D). Importantly, compared with the NC agomir group and the vein graft group, a significant reduction in PCNA-positive cells could be induced in the neointima after local delivery with miR-126-3p agomir (13.17 ± 1.64% vs. 24.83 ± 2.26% and 23.67 ± 2.35%, respectively, *P* < 0.01, Figure 7H).

### Overexpression of miR-126-3p by agomir improved reendothelialization *in vivo*

**Figure 8:**
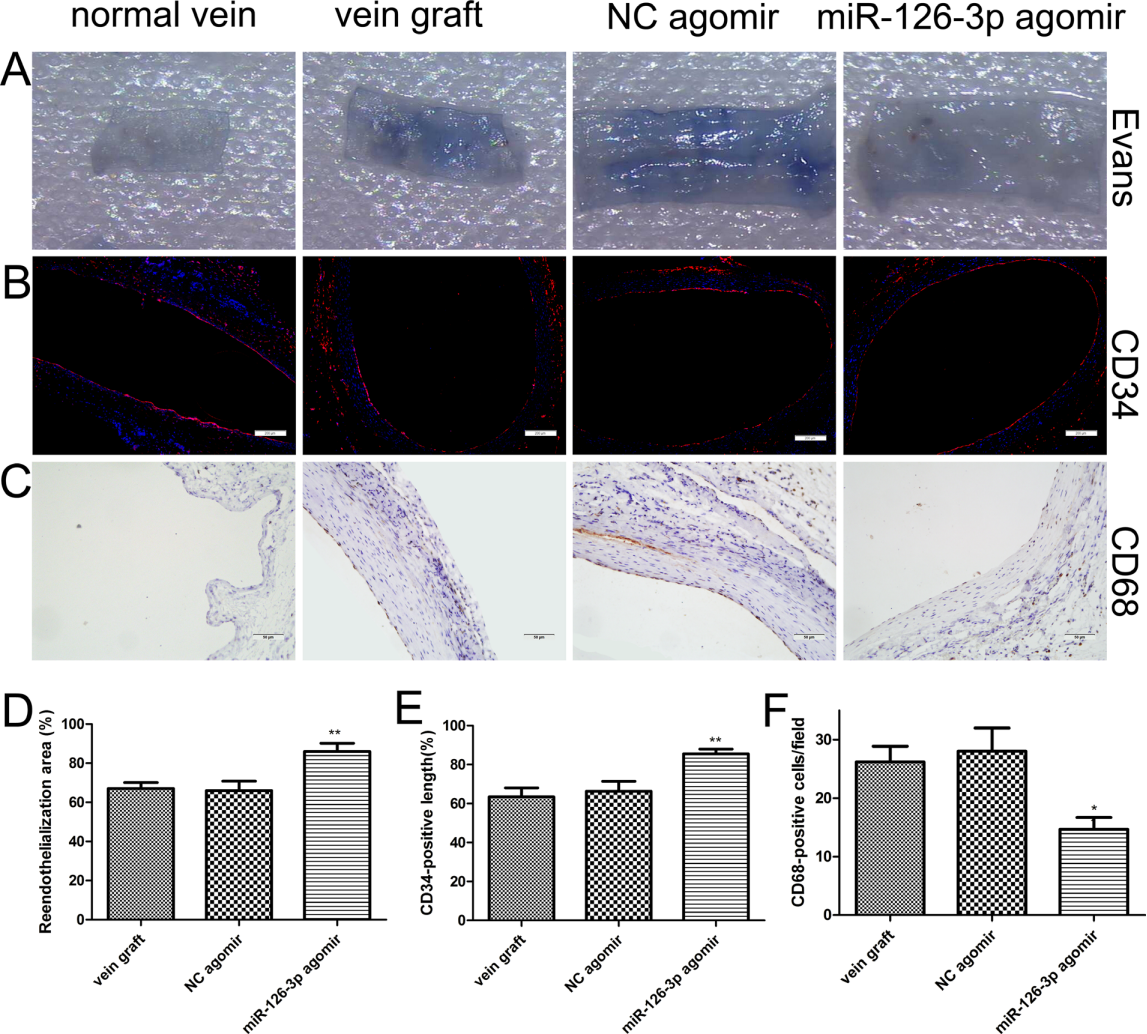
miR-126-3p agomir promoted reendothelialization *in vivo* ***(*A**) Representative Evans Blue staining photomicrographs of vessel wall harvested from normal and vein arterialization rats at 14 days after surgery. (**B**) Immunofluorescence staining for vascular endothelial cell marker CD34 (red) was used to assess endothelial recovery of the vein graft for each group. Nuclei were stained with DAPI (blue). Scale bars represent 200 μm. (**C**) Representative staining of CD68 within the intima layer 28 days after surgery is shown. Scale bars represent 50 μm. (**D**) The ratio of non-stained area (white) to the total en face area of the vein graft was used to evaluate reendothelialization. (**E**) Quantification of the reendothelialized areas was performed as a percentage of the CD34-positive surface to the total luminal surface (**F**) Quantitative evaluation of the number of accumulating CD68-positive monocytes/macrophages within the vascular wall. Values are expressed as the mean ± SEM. *n* =6 per group. *P < 0.05, **P < 0.01vs NC agomir and vein graft groups.

We sought to determine whether the beneficial effects of the miR-126-3p agomir were associated with improved reendothelialization in the vein grafts. Evans blue staining was performed in the vein grafts at 14 days after surgery. Luminal staining for Evans blue indicated that reendothelialization was significantly greater in the miR-126-3p agomir group in comparison with the NC agomir group and vein graft group (86% ± 4.19% vs. 66% ± 4.9% and 67.17% ± 2.99%, respectively, *P* both < 0.01, Figure 8A and 8D). Furthermore, we also specifically analyzed the extent of reendothelialization by using the EC surface marker CD34 at day 14 after surgery. Quantitative analysis of immunopositive cells in the lumen of vein grafts revealed that reendothelialization was significantly increased in the miR-126-3p agomir group and reached 85% by day 14. Moreover, reendothelialization in the NC agomir group and the vein graft group was only approximately 65% (85.5% ± 2.53% vs. 66.33% ± 5.16% and 63.5% ± 4.62%, respectively, *P* both < 0.01, Figure 8B and 8E). As noted above, an integrated endothelial cell barrier prevents intimal inflammatory cell infiltration to limit VSMC proliferation and neointimal development. Therefore, we also performed CD68 immunohistochemical staining under the assumption that the infiltration of inflammatory cells into the vein grafts might be increased owing to the incomplete endothelialization. As shown in Figure 8C, the normal vein showed no evidence of positive staining, while the grafted veins showed a marked increase in CD68-positive monocyte/macrophage staining at 28 days after surgery (Figure 8E). However, miR-126-3p agomir treatment resulted in a significant decrease in cell infiltration compared with the NC agomir group and vein graft group (14.67 ± 2.04 vs. 28 ± 4 and 26.17 ± 2.7 cells/field, *P* both < 0.05, Figure 8F). All these results demonstrated that, consistent with observations *ex vivo*, the miR-126-3p agomir delivery locally accelerates reendothelialization after venous implantation and thereby attenuates vein graft luminal stenosis and neointimal formation.

## DISCUSSION

In the past decade, robust studies in the miRNA field have provided appealing evidence in support of the role of miRNAs in cardiovascular function and disease, and shown great potential clinical applications [31, 32]. Given the recent demonstration that miRNA-based therapeutic approaches, MRX34 and Miravirsen, have successfully reached phase I and II clinical trials [33, 34], one may speculate that it is only a matter of time before miRNA-based drugs are identified and validated for the treatment of vascular diseases.

In principle, miRNA mimics and inhibitors are synthetic oligonucleotides that elevate the expression of beneficial miRNAs or suppress the pathogenetic miRNA by sequence complementarity. However, naked oligonucleotides are less efficient due to their instability *in vitro* or *in vivo*, which subject them to different nucleases [31]. Thus, various delivery tools for miRNA-based therapeutics have been developed to enhance gene delivery, such as viral vectors, lipid-based vehicles, and cationic polymers. Viral systems have substantially advanced the field of gene therapy because of their high transfection efficiency. However, there are several limitations, including carcinogenesis and broad immunogenicity tropism. Non-viral methods have the potential to address many of these limitations, particularly with respect to safety. However, poor cellular uptake, rapid degradation by nucleases, and limited blood stability are disadvantages [35, 36]. As a result of these limitations, diversified chemistry modifications of oligonucleotides have been designed to address these problems. agomirs are one type of chemically modified miRNAs that possess advantages, such as improved cellular uptake, a long half-life, a weak immune response, high biocompatibility, and increased stability in cells [36]. In the present study, the miR-126-3p agomir was used because of its excellent prospects in clinical applications relative to viral vectors.

Previous studies have shown that miRNAs are abundant and relatively stable in the circulation, suggesting that they may play a paracrine role in controlling gene expression [37]. Several groups have revealed that miR-126-3p levels are low in the serum of stable CAD patients, and there is a positive association between circulating miR-126 and myocardial infarction [20, 38]. Olivieri, F. et al. demonstrated that plasma loss of endothelial miR-126-3p in patients with type 2 diabetes is associated with diabetic vascular complications [21]. In our study, all of these severe CAD patients who were preparing for CABG had a significantly lower level of miR-126-3p expression compared with the healthy subjects. This result is in agreement with the results of earlier studies that the endothelial cell-enriched and vasculoprotective miR-126-3p level was significantly lower in patients with stable CAD [38]. Moreover, we found that circulating miR-126-3p levels showed a brief rise in the perioperative period. The result may be explained by the idea that the change may reflect an early compensatory mechanism by which endothelial cell function is maintained in vein grafts. This idea is supported by a previous report that miR-126-3p was released from activated endothelial cells by apoptotic bodies and served as a compensatory signal to confer atheroprotection in atherosclerosis [39]. Our data for the HSV organ cultures *ex vivo* also support this hypothesis. Our results established the basic clues demonstrating that miR-126-3p participates in CABG. It must be noted that this study has relatively small blood sample size and should be corroborated by subsequent studies.

A main highlight of our study is the choice and use of the exact cells that are dysfunctional in graft failure following CABG in patients with CAD, namely, HSVECs and HSVSMCs. Improvement of endothelial function is a significant therapeutic goal in vein graft disease. It is commonly recognized that the proliferative and migratory activities of ECs play a central role in the promotion of reendothelialization and restoration of EC function [8, 9]. Among the verified targets of miR-126-3p, PIK3R2 and SPRED-1 are known as negative regulators of the VEGF signaling pathway to critically influence proliferation and migration in human umbilical vein endothelial cells (HUVECs) [24]. However, adult HSVECs showed a very significant phenotypic and functional differences compared with HUVECs because of the different origin of cells and exposure settings [40]. Here, for the first time, we identified the role of miR-126-3p in the proliferation and migration of HSVECs using gain- and loss-of-function studies. We newly confirmed that miR126-3p targets SPRED-1 and PIK3R2 and activates the ERK1/2 and AKT signaling pathways in HSVECs. This finding is in agreement with previous reports demonstrating the activation of the ERK1/2 and AKT signaling pathways in VEGF stimulation of endothelial cell proliferation and migration [29]. Moreover, it is possible to manipulate miR-126-3p expression and observe changes in target gene expression by the agomir method.

The damaged vessel wall is reliant on endothelial cell proliferation and migration to promote reendothelialization and preserve EC function, thereby limiting neointimal development. By contrast, subendothelial SMC proliferation and migration stimulated by inflammation are major contributors to neointimal development [6, 41]. Interestingly, miR-126-3p has emerged as a controversial miR involved in SMC proliferation and migration. Jansen et al. found that intercellular transfer of miR-126-3p reduces arterial SMC proliferation and migration, which was contrasted with the experimental results that decreased expression of miR-126-3p led to increased proliferation of arterial SMCs by Zhou et al [27, 42, 43]. Hence, we considered worth to further investigating dysregulated miR-126-3p expression on migration and proliferation in HSVSMCs before moving to translational studies. Unexpectedly, but within our understanding, we found that overexpression of the miR-126-3p by agomir had no effect on migration and proliferation in HSVSMCs. This result is consistent with previous reports showing that the function of EC-specific miR-126-3p on cell migration and proliferation is cell type-specific [28]. This property differs from that of many other miRNAs, as miR-135b-5p and miR-499a-3p, for example, not only promote cell proliferation and migration in ECs but also in VSMCs [44].

An organ culture of HSVs closely mimics the intimal hyperplasia that occurs *in vivo* after vein graft transplantation. The most obvious feature of this model is that it not only allows much better control and monitoring of chemical and mechanical environments than that permitted *in vivo* but also provides a useful experimental system to study whole-vessel behavior that is not feasible in cell culture [45, 46]. Using this model, we have demonstrated that miR-126-3p expression can be effectively manipulated by agomir to accelerate reendothelialization and reduce neointimal hyperplasia in clinically relevant vein grafts. However, consistent with the majority of studies, our data regarding neointimal formation were derived from an organ culture model with several limitations, such as the absence of hemodynamic alterations in the arterial circulation and of infiltrating inflammatory cells and other circulating blood components, which contribute to the development of intimal hyperplasia *in vivo* [16, 47]. Hence, the translational application of our findings requires further validation in *in vivo* experiments.

To our knowledge, only a few *in vivo* studies have attempted to illuminate the role of miR-126-3p in the development of intimal hyperplasia in various vessels, and none have studied vein grafts. Two different groups confirmed that Ad-mediated overexpression of miR-126-3p and endothelial microparticle-mediated transfer of miR-126-3p in balloon-injured arteries promoted reendothelialization in arterial balloon injury models [26, 28]. Recent research has provided evidence that miR-126-3p NP-conjugated stents significantly inhibit the development of neointimal hyperplasia in a rabbit model of endothelial denudation of the iliac artery [27]. By contrast, Zhou et al. reported that systemic depletion of miR-126-3p in mice inhibited neointimal lesion formation of carotid arteries induced by cessation of blood flow [43]. Given the controversial results present in various models, we aimed to clarify the effects of the miR-126-3p agomir in an established rat vein arterialization model as described [48, 49]. As it known to all that CABG provides a practical opportunity for the local delivery of agents that can regulate the pathophysiology of autologous vein graft restenosis inside the operating room, we propose that the upregulation of miR-126-3p levels in a rat vein arterialization model at the time of engraftment through *ex vivo* treatment of the venous wall is a clinically acceptable administration method that could lead to localized manipulation of the target miRNA. This finding is another highlight of our study. Fluorescent staining showed that local application of the miR-126-3p agomir was effective, long-lasting, and restricted to the conduit, and it did not affect other organs. Furthermore, the results were confirmed by western blot analysis. We measured the diameter and the blood flow of vein grafts via vascular ultrasound and found that the diameter was wider and the blood flow was lower in the miR-126-3p agomir-treated group compared with the other groups. These data were consistent with the results obtained using a miR-221 sponge-based therapy in rat vein grafts [17]. Moreover, hematoxylin eosin staining revealed that treatment with the miR-126-3p agomir dramatically reduced neointimal thickness, supporting earlier observations in balloon-injured arteries in rats and rabbits [27, 28]. In addition, Masson trichrome and immunohistochemical staining of α-SMA and PCNA provided findings consistent with previous reports that the thickening of the neointima is characterized by deposition of VSMCs and ECM components [6]. Our results strengthen hypothesis that local delivery of miR-126-3p by agomir at the time of engraftment is sufficient to bind target sequences and suppress neointimal formation in vein grafts *in vivo*.

It has been reported that the ability of the endothelium to repair is dependent on both the proliferation and migration of the remaining intact endothelial cells and the recruitment of the body's own circulating endothelial progenitor cells (EPCs) derived from the bone marrow, which in turn differentiate into endothelial phenotypes, although the role of this EPC has been a controversial topic. However, in our previous studies, the systematic administration of exogenous stems cells has played a role in the preservation of endothelial function and inhibition of neointimal formation [7, 48]. In the present study, our results showed that miR-126-3p overexpression in the rat vein grafts accelerated reendothelialization. We speculate that the therapeutic effect may be due to the proliferation and migration of surrounding resident endothelial cells. However, whether circulating EPCs were really involved in the therapeutic effects of the miR-126-3p agomir requires further investigation.

In summary, we have demonstrated that miR-126-3p promotes proliferation and migration in HSVECs and that transfection of the miR-126-3p agomir significantly improves reendothelialization and thus prevents neointimal formation of the vein graft *ex vivo* and *in vivo*. To the best of our knowledge, this is the first report to demonstrate that miR-126-3p exerts a vasculoprotective role in the setting of vein graft disease. The protective mechanisms may involve strengthening of the ERK1/2 and AKT signaling pathways via the suppression of SPRED-1 and PIK3R2 expression. We have provided strong evidence that the mRNA-126-3p agomir possesses potential clinical value for the prevention and treatment of autologous vein graft restenosis in CABG.

## MATERIALS AND METHODS

### Ethics statement

The present study was conducted in accordance with the principles of the Declaration of Helsinki and approved by the Ethical Committee of Qilu Hospital of Shandong University (KYLL-2017-105, Jinan, China). Written informed consent was obtained from all subjects before entering the study. Experiments involving live animals were conducted according to the guidelines of the Animal Care and Use Committees at Qilu Hospital of Shandong University.

### Study population and blood collection

Ten patients undergoing elective CABG in the department of cardiovascular surgery of our hospital were enrolled in this study. All of them had a history of chest pain, characteristic electrocardiogram changes and the diagnostic coronary angiography results showed triple-vessel disease that was not suitable for stenting. In addition, ten healthy volunteers were enrolled as a control group at the medical examination center of our hospital. There were no significant differences in the baseline characteristics of the two groups (Supplementary Table 1). Peripheral blood samples (2 ml) were collected from the patients into EDTA tubes 1 day before and 1, 3, 7 and 14 days after surgery, and only one blood sample was collected from each person in the control group. Venous blood samples were processed within 1 h of collection by two-step centrifugation as described [50]. After centrifugation, the supernatant was transferred to RNase-free tubes and maintained at –80°C until RNA extraction.

### Cell isolation and culture

Discarded HSV segments were obtained from patients receiving CABG surgery in our hospital. HSVECs were isolated from HSV segments by standard collagenase digestion as described previously [51, 52]. The endothelial phenotype was confirmed by the typical cobblestone and nonoverlapping appearance and by positive staining for von Willebrand factor (vWF). HSVECs were maintained in ECM medium supplemented with 25 ml of fetal bovine serum (FBS), 5 ml of endothelial cell growth supplement and 5 ml of penicillin/streptomycin solution. HSVSMCs were obtained by the explant method as previously described [53]. The VSMC phenotype was confirmed by the typical “hill and valley” growth pattern in culture and by immunofluorescence staining using antibodies against α-smooth-muscle actin (α-SMA). HSVSMCs were cultured in DMEM containing 25 mmol/l glucose and 10% FBS. For this study, all cells were kept in a humidified incubator maintained at 37°C and supplied with 5% CO2 and 95% air, and cells from the third to seventh passages were used for the experiments.

### Transfection

miR-126-3p agomir and miR-126-3p antagomir were obtained from RiboBio (Guangzhou, China) and transfected into cells using riboFECTTM CP transfection reagent (RiboBio) according to the manufacturer’s protocol. Following an incubation period of 24–48 h, the cells were harvested and functional analysis was performed. Since agomir-miRNA is a chemically modified miRNA mimic, PCR detection methods are unable to directly detect the change in information for the target miRNA. Thus, we tested the transfection efficiency by using a Cy3-labeled miR-126-3p agomir, and visualized the results using a fluorescence microscope.

### *In vitro* proliferation and migration assays

To examine the proliferation rates of cells, an EdU incorporation assay was performed with the Cell-Light™ EdU Apollo^®^567 *In Vitro* Imaging Kit (RiboBio) according to the manufacturer’s specifications. Briefly, agomir-transfected cells in 24-well plates were incubated with 20 μM EdU for 2 hours before fixation, permeabilization, and EdU staining. Subsequently, cell nuclei were stained with Hoechst 33342 and examined under a fluorescence microscope. The proliferation rate was defined as the percentage of EdU-stained cells to Hoechst 33342-stained cells.

To assess cell migration *in vitro*, a scratch wound assay was performed as described with minor modifications [54]. Briefly, the wounds were produced by a sterile 200-µl pipet tip 24 hours after treatment, and the cells were cultured in serum-free medium to eliminate the effect of cell proliferation. The marked position were examined and photographed with a microscope at 0 and 24 hours post-scratch time points. The numbers of cells that migrated into the wound fields were counted, and further analysis was performed.

Cell migration was further assessed using Transwell chambers with 8-mm pores (Corning, Lowell, USA) as described previously [48]. Briefly, after 24 hours of transfection with agomirs, the cells were suspended in serum-free medium at an optimized concentration and placed in the upper chamber of the Transwell. The chamber was then transferred to a well containing complete medium. The membranes were allowed to migrate for 24 hours and then stained with crystal violet dye. The cells in the top well were removed, and the remaining cells were counted in at least 3 random microscopic fields per well.

### Human saphenous vein organ culture

Organ cultures were performed using distended HSVs essentially as described previously [45, 46]. Each fragment of HSVs was obtained from patients undergoing CABG surgery in our hospital and cut into 0.5-cm rings. For the PCR experiment, three sequential rings were cultured with RPMI 1640 medium supplemented with 30% fetal bovine serum and L-glutamine for a period of 7 and 14 days. To explore the effect of the miR-126-3p agomir in HSV organ culture, four equal individual segments were created for each leftover HSV, one segment (uncultured control) was immediately formalin-fixed, and the remaining segments were cultured in complete medium. In two of the three cultured segments, miR-126-3p agomir and miR-126-3p negative control agomir (NC agomir) were transfected for the initial culture; the remaining segment received no treatment (untreated control). The medium were changed every 2 days, and the veins were embedded in paraffin, sectioned, and stained with hematoxylin-eosin (HE) and endothelial cell marker 14 days after culture.

### Rat vein graft model and experimental groups

An established local delivery rat vein graft model was performed as previously described [48, 49]. In brief, adult male Wistar rats weighing 250 to 300 g were anesthetized with chloral hydrate by intraperitoneal injection and systemically heparinized. Next, a 20-mm segment of the right external jugular vein was excised, the infrarenal abdominal aorta was liberated, and the right external jugular vein was autologously inserted into the abdominal aorta using a 20-GA intravenous cannula (BD, Sweden) with the cuff technique. During surgery, 5 nmol miR-126-3p agomir or miR-126-3p negative control agomir (NC agomir), dissolved in normal saline (0.9%) according to the manufacturer’s instructions, was perfused into the vein graft under a distending pressure of 20 mmHg for 10 minutes at room temperature before arteriovenous anastomosis.

Four groups were created after grafting: (1) normal vein group, *n* = 21; (2) vein graft group (vein grafted to the abdominal aorta without transfection), *n =* 21; (3) miR-126-3p negative control agomir group (NC agomir group, vein grafts treated with miR-126-3p negative control agomir), *n =* 21; (4) miR-126-3p agomir group (vein grafts treated with miR-126-3p agomir), *n =* 27. The efficiency of agomir transduction was evaluated by fluorescent light examination of frozen vascular sections using a Cy3 -labeled miR-126-3p agomir at 1 and 7 days after surgery, and further confirmed by western blot analysis at 7 days after surgery. To detect reendothelialization, six rats from each group received an intravenous injection via the tail vein of 1.5 ml 0.5% Evans blue dye 30 minutes before euthanization, and another six rats from each group were euthanized directly for CD34 immunofluorescence staining 14 days after grafting. All the left rats were humanely killed 4 weeks after vein grafting, and special staining and immunohistochemical staining of paraffin sections of vein grafts were performed for histological examination.

### Vascular ultrasound examination of vein grafts

We performed vascular ultrasound examination of vein grafts in live rats using a small animal ultrasound scanner (Vevo2100, Toronto, Canada) operated by an experienced ultrasonic engineer. Rats were anesthetized using chloral hydrate and fixed on a mobile platform. Signs of perioperative thrombus formation were carefully checked during the whole observation period. The luminal diameter and blood flow in the distal anastomosis of vein grafts were measured using a linear array transducer (MS210) at day 28 before tissue harvesting.

### HE staining and Masson trichrome staining

For histological examination, vein grafts were fixed in 4% paraformaldehyde, embedded in paraffin, and cut into 5-μm-thick sections. Sections of vein grafts were stained with HE to measure the neointimal thickness, and sections of rat vein grafts were also stained with Masson trichrome to detect collagen. We did not the distinguish intima and media in the arterialized vein because both of them thickened significantly without any morphologic border. Image analysis software was used to quantify the neointimal thickness and collagen content.

### Immunohistochemical staining

Immunohistochemical staining was carried out using SP-9100 Detection Kits (ZSGB-BIO, Beijing, China) according to the manufacturer’s protocol. Briefly, paraffin-embedded sections of vein grafts were incubated with primary antibodies against CD31 (1:200, Abcam, Cambridge, USA), α-smooth-muscle actin (α-SMA 1:200, Abcam), proliferating cell nuclear antigen(PCNA,1:1000, Abcam),and CD68 (1:200, BOSTER, Wuhan, China) overnight at 4°C, followed by incubation with secondary antibodies for 15 min at room temperature. The sections were developed in DAB solution and then counterstained with hematoxylin. Measurements for histological parameters (reendothelialization, α-SMA positive areas, percentage of proliferating cells, and macrophage count) were performed by one of the authors who was not informed about the group assignment.

### Immunofluorescence

For immunofluorescence staining, vein grafts were fixed in 4% paraformaldehyde, dehydrated in 20% and 30% sucrose, embedded in OCT compound, and cut into 10-μm-thick sections. Tissue sections were blocked with 5% goat serum for 1 h at room temperature and incubated with rabbit anti-CD34 (1:200, Abcam) overnight at 4°C. Subsequently, the sections were stained with secondary rhodamine–conjugated rabbit anti-goat antibody (Proteintech, Wuhan, China) and counterstained with DAPI. To assess reendothelialization, the percentage of the CD34-positive area to the whole luminal surface was measured as described [55]. For cell immunofluorescence to identify primary cells isolated from HSVs, anti-vWF-FITC (1:100, Abcam) and anti-α-SMA (1:200, Abcam) antibodies were used.

### Western blot analysis

Cells and tissues were harvested and lysed in RIPA buffer using established methods. Equal amounts of proteins were resolved by SDS-PAGE and transferred to PVDF membranes. Blots were incubated with the appropriate primary antibodies, including PIK3R2 (1:10000, Abcam), SPRED-1 (1:10000, Abcam), AKT(1:1000, Cell Signaling, Boston, USA), p-AKT (1:1000, Cell Signaling), ERK1/2 (1:1000, Cell Signaling), p-ERK1/2 (1:1000, Cell Signaling), and GAPDH (1:1000, Proteintech), followed by incubation with HRP-conjugated secondary antibodies and protein visualization by chemiluminescence using ECL Substrate according to the manufacturer’s instructions. Densitometric analyses were performed using Image-J software.

### RT-PCR for miRNA and mRNA

Total RNA was isolated using RNA Isolation Kits (Takara, Dalian, China), and levels of miR-126-3p were measured using the All-in-One™qRT-PCR Detection System (Genecope, Rockville, USA) according to the manufacturer’s specifications.

Cel-miR-39 was used as an endogenous control for plasma samples, and U6 was used as the endogenous control for tissue samples. Expression levels of PIK3R2 and SPRED-1 were measured using a ReverTra Ace q-PCR RT Kit (Toyobo, Osaka, Japan) following the manufacturer’s instructions, and GAPDH was used for normalization. The primer sequences are listed in Supplementary Table 2. The relative expression levels between the groups was calculated using the ∆∆Ct method as we have previously desc ribed [48].

### Statistical analysis

All statistical analyses were performed with SPSS19.0 software. The data are expressed as the mean ± SEM. Statistical comparisons were conducted with Student’s *t*-test and one-way ANOVA followed by the Student-Newman-Keuls (SNK) test. *P* < 0.05 was considered significant. All experiments were repeated at least three times.

## SUPPLEMENTARY MATERIALS FIGURES AND TABLES


