# Supplementary material for: Upregulation of miR-126-3p promotes human saphenous vein endothelial cell proliferation in vitro and prevents vein graft neointimal formation ex vivo and in vivo
Source: Oncotarget. 2017 Nov 3;8(63):106790–806. doi: 10.18632/oncotarget.22365 (PMC5739774; doi:10.18632/oncotarget.22365)
Supplement: Supplementary file 1 [file oncotarget-08-106790-s001.pdf]

Upregulation of miR-126-3p promotes human saphenous vein endothelial cell proliferation *in vitro* and prevents vein graft neointimal formation *ex vivo* and *in vivo*

SUPPLEMENTARY MATERIALS

Supplementary Table 1: Clinical data on CABG patients and healthy subjects

Parameters	CABG patients (*n* = 10)	Healthy subjects (*n* = 10)	*P* value
Age, median (range) , y	4 (49–74)	5 (50–68)	*P* = 0.1704
Gender, male, *n* (%)	4 (40%)	5 (50%)	*P* = 0.6733
Body mass index (kg/m2)	26.06 ± 1.24	26.44 ± 4.26	*P* = 0.7905
Alcohol, *n* (%)	4 (40%)	3 (30%)	*P* = 0.6601
Smoker, *n* (%)	3 (30%)	4 (40%)	*P* = 0.6601
Family history, *n* (%)	3 (30%)	2 (20%)	*P* = 0.6278
Hypertension, *n* (%)	4 (40%)	1 (10%)	*P* = 0.1346
Diabetes, *n* (%)	3 (30%)	1 (10%)	*P* = 0.2878

Supplementary Table 2: The primers for real-time PCR

Primer of target	Sequence (5′ to 3′)
PIK3R2 forward primer	ACCCGTGTAATTGGACATAGGA
PIK3R2 reverse primer	GCTTCCATTTTTACTTTCTCTTCCA
SPRED-1 forward primer	CGAGATGGGCACTAGGCTT
SPRED-1 reverse primer	ACTCGTGGCGGTAGTGATTG
GAPDH forward primer	GCACCGTCAAGGCTGAGAAC
GAPDH reverse primer	TGGTGAAGACGCCAGTGGA
miR-126-3p	UCGUACCGUGAGUAAUAAUGCG
miR-126-5p	CAUUAUUACUUUUGGUACGCG
U6	ATTGGAACGATACAGAGAAGATT
Cel-miR-39	TCACCGGGTGTAAATCAGCTTG

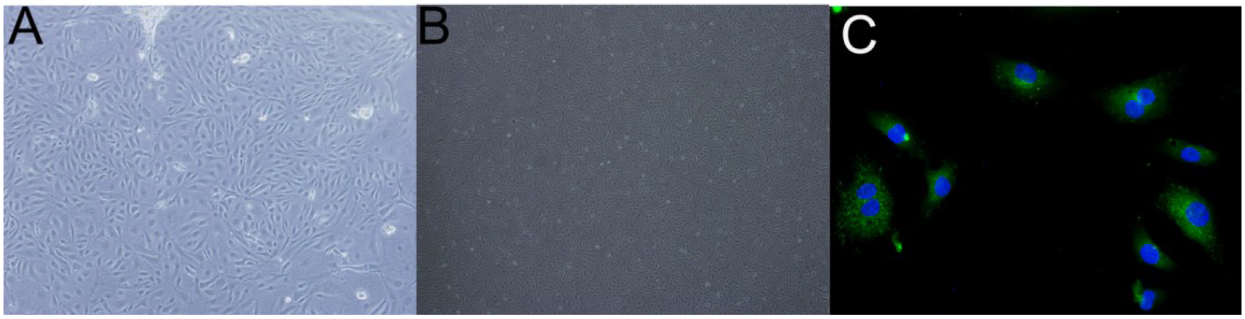

Supplementary Figure 1: Characteristics and identification of HSVECs. (**A**) HSVECs cultured for 10 days were mainly characterized by a round and spindle-like shape (×100). (**B**) HSVECs cultured for 3 passages exhibited a cobblestone and nonoverlapping appearance (×40). **(C**) HSVECs were identified by immunofluorescence staining for von Willebrand factor (vWF), a mature endothelial cell marker. Nuclei were stained blue with DAPI (×400)

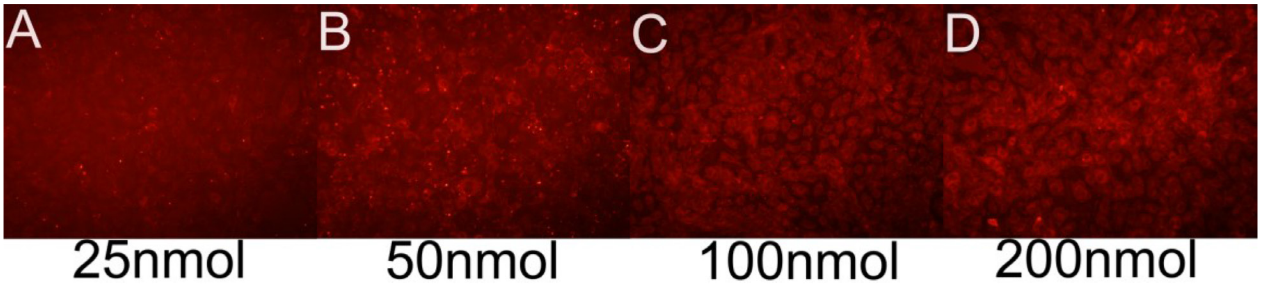

Supplementary Figure 2: To demonstrate the transfection efficiency of the agomir, cells were transfected with 25, 50, 100 or 200 nmol of Cy3-labeled miR-126-3p agomir for 1 day. Representative fluorescence microscopy image demonstrating cytoplasmic localization of the fluorescent agomir after chemical transfection in EC (×100). (**A**) 25 nmol; (**B**) 50 nmol; (**C**) 100 nmol; and (**D**) 200 nmol.

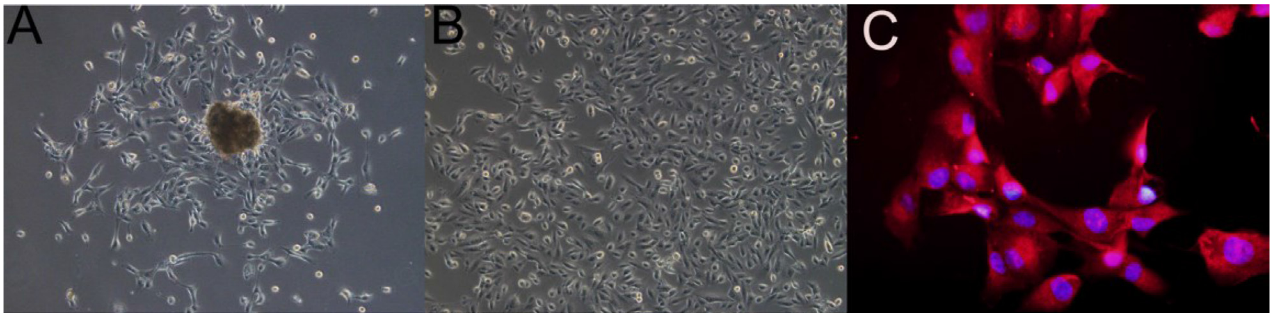

Supplementary Figure 3: Characteristics and identification of HSVSMCs. (**A**) HSVSMCs were observed to migrate out from the explants at 7 days (×100). (**B**) HSVSMCs cultured for three passages exhibited a typical colony, characterized by the typical "hill and valley" growth pattern (×100). (**C**) HSVECs were identified by immunofluorescence staining for α-smooth-muscle actin (α-SMA). Nuclei were stained blue with DAPI (×400).

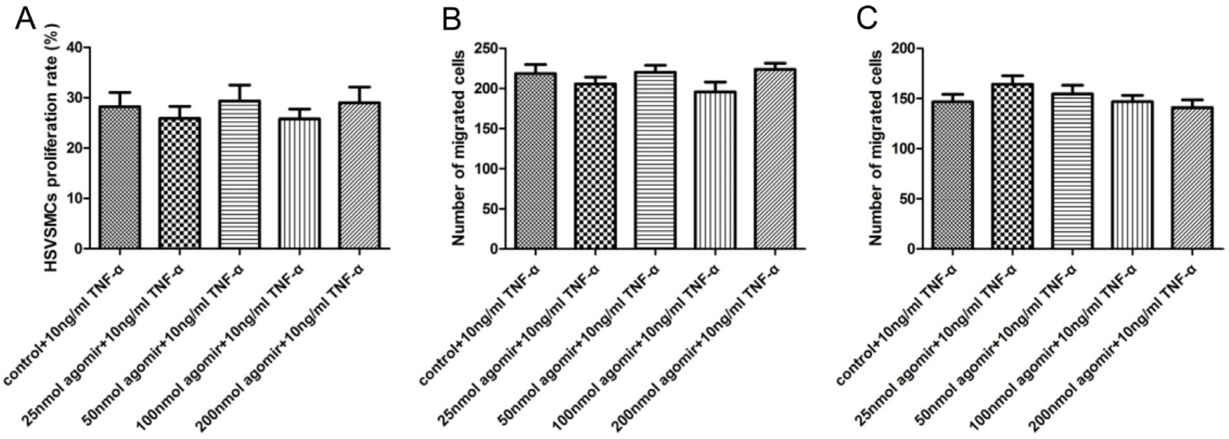

Supplementary Figure 4: miR-126-3p agomir had no effect on TNF-α-induced HSVSMC proliferation and migration *in vitro*. (**A**)The results of the EdU incorporation assay showed that increasing concentrations of miR-126-3p agomir had no effect on TNF-α-induced proliferation. (**B**) The results of the scratch wound assay showed that increasing concentrations of miR-126-3p agomir had no effect on TNF-α-induced migration. (**C**) The results of the transwell assay showed that increasing concentrations of miR-126-3p agomir had no effect on TNF-α-induced migration.

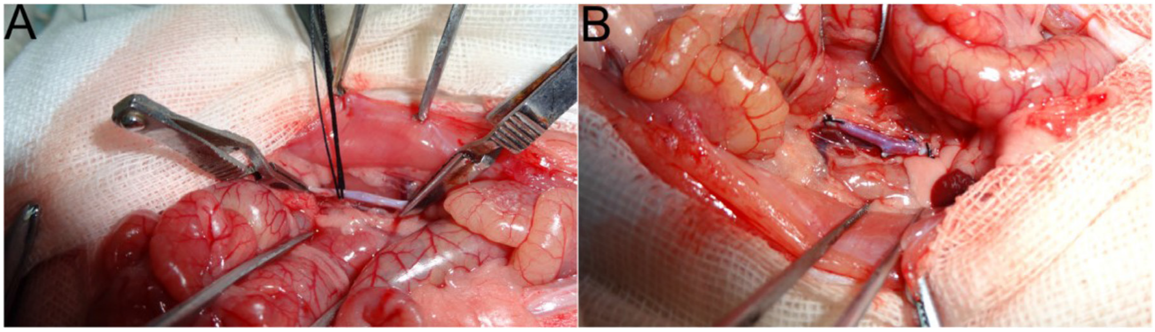

Supplementary Figure 5: Rat vein graft model. (**A**) The abdominal aorta was separated as shown in the photograph. (**B**) The external jugular vein graft was successfully implanted into the infrarenal aorta using a "cuff" anastomotic technique after transfection, and the vein graft was filled well.

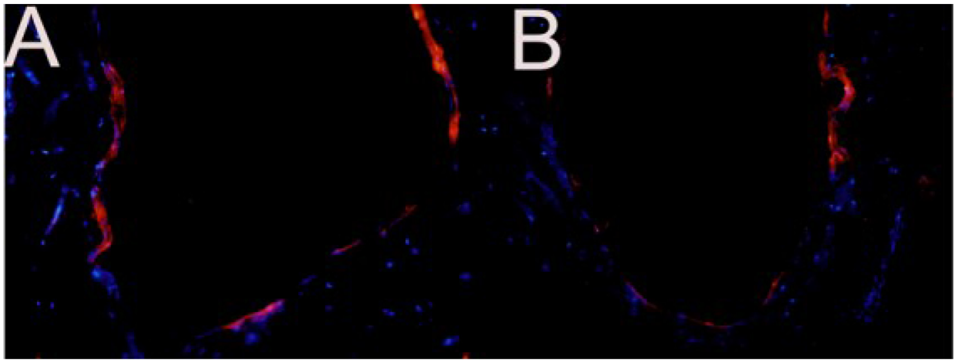

Supplementary Figure 6: miR-126-3p agomir transfection in vivo. (**A**) At 24 hours after grafting, Cy3-labeled agomir (red) was localized in almost all endothelial cells by fluorescence microscopy (×200). (**B**) Red fluorescence was still detectable in cells of the vascular walls at 7 days after transfection (×200).
